# Artificial Intelligence-Assisted Longitudinal Analysis Reveals a Novel Rare Fainting Wisdom Tooth Phenomenon in Fully Dentate Mandibles

**DOI:** 10.1155/ijod/1932229

**Published:** 2025-10-12

**Authors:** Rellyca Sola Gracea, Shivi Chopra, Peter Östgren, Daniel Benchimol, Reinhilde Jacobs

**Affiliations:** ^1^OMFS-IMPATH Research Group, Department of Imaging and Pathology, Faculty of Medicine, KU Leuven, Leuven, Belgium; ^2^Department of Oral and Maxillofacial Surgery, University Hospitals Leuven, Leuven, Belgium; ^3^Department of Dentomaxillofacial Radiology, Faculty of Dentistry, Universitas Gadjah Mada, Yogyakarta, Indonesia; ^4^Department of Dental Medicine, Karolinska Institutet, Stockholm, Sweden; ^5^Department of Oral and Maxillofacial Radiology, Folktandvården Stockholm, Stockholm, Sweden

**Keywords:** artificial intelligence, mandible, panoramic radiograph, third molar, tooth impaction, wisdom tooth

## Abstract

**Objectives:**

This study aims to characterise the rare 'fainting wisdom tooth' phenomenon, defined as the angular transition of a mandibular wisdom tooth from an initially upright position to mesial or horizontal inclination during adolescence in fully dentate mandibles.

**Materials and Methods:**

A retrospective longitudinal analysis was conducted using panoramic radiographs from a pooled database of 11,932 patients. Fifty cases of fainting wisdom teeth (0.4% prevalence) were identified using a validated deep learning (DL) convolutional neural network (CNN)-based artificial intelligence (AI) tool and compared to 50 matched controls with vertically erupted wisdom teeth. Radiographic variables were measured at two time points: T1 (ages 8–15) and T2 (ages 16–23). Angular changes and anatomical features, including space/crown width ratio, tooth depth, proximity to the second molar and mandibular canal, cemento-enamel junction (CEJ)/apical width ratio and gonial angle, were assessed. Logistic regression was used to determine predictive factors associated with the fainting phenomenon, with results reported as odds ratios (ORs).

**Results:**

Fainting wisdom teeth showed a significant angular shift from a median of 29° at T1 to 83° at T2, while controls showed minimal change. Logistic regression identified tooth depth as the strongest predictor (OR = 0.1, *p* < 0.001), with additional risk factors including reduced space/crown width ratio, increased proximity to the second molar, higher CEJ/apical width ratio and increasing gonial angle.

**Conclusion:**

This is the first study to describe and analyse the rare fainting wisdom tooth phenomenon using AI-assisted longitudinal analysis of panoramic radiographs. The findings suggest that early tooth positioning and surrounding anatomical constraints may interfere with vertical eruption and increase the risk of fainting.

## 1. Introduction

Third molars, commonly referred to as wisdom teeth, are notorious for their tendency to become impacted within the jaw [1]. Impaction occurs when these teeth fail to fully erupt into their normal position due to lack of space or obstruction within the dental arch. Among all teeth, mandibular wisdom teeth exhibit the highest rate of impaction, presenting significant clinical challenges and potential complications for individuals [2].

Impacted wisdom teeth can lead to complications, including pain, swelling, infection and damage to adjacent teeth. In severe instances, they may also contribute to the development of periodontal disease, cysts or tumours. The management of impacted wisdom teeth often involves surgical intervention to remove the affected teeth and alleviate associated symptoms [3]. To mitigate these risks, early evaluation or timely monitoring of wisdom tooth development during adolescence is crucial, as it allows clinicians to define appropriate preventive strategies, whether through early extraction or orthodontic treatment planning. Such early intervention can significantly reduce the likelihood of complications associated with late-stage impactions [4].

To support preventive efforts and enhance early diagnosis, numerous studies have been conducted to elucidate the factors that influence its occurrence. Researchers have sought to identify predictive markers and risk factors associated with impaction, in order to improve early detection and preventive measures using artificial intelligence (AI)-based tools [5–7]. Within the broader field of AI, deep learning (DL) models such as convolutional neural networks (CNNs) are the preferred choice for image-based dental tasks. CNNs have enabled automated segmentation and angulation measurement of mandibular wisdom teeth on panoramic radiographs. These DL-based systems provide clinicians with valuable decision-support tools during adolescent eruption monitoring [7].

However, despite these efforts, it has been observed that wisdom teeth may exhibit changes in angulation over time, adding complexity to existing prediction models [8–10]. Longitudinal analysis of panoramic radiographs revealed a rare eruption pattern, characterised by the transition of an initially upright mandibular third molar to a horizontal inclination during adolescence, despite the presence of a fully dentate mandible. This pattern, denoted as the 'fainting wisdom tooth' phenomenon, represents an exceptional deviation from expected eruption trajectories and, to our knowledge, has not been systematically described in the literature. Although uncommon, this presentation may result in significant clinical consequences if not identified early.

Therefore, the present study aimed to investigate factors influencing the likelihood of wisdom teeth transitioning into fainting molars in fully dentate mandibles. Specifically, this study compared panoramic radiographs over time, using an AI-assisted tool to assess mandibular molar angular changes and identify mandibular wisdom teeth transitioning from an initial upright position to significant horizontal mesial tilting, referred to as the 'fainting wisdom tooth' phenomenon. Identifying the risk of angular changes could improve clinical decision-making for timely intervention, potentially preventing impaction and reducing the risk of complications.

## 2. Materials and Methods

This retrospective longitudinal study employed panoramic radiographs from the clinical database of the Public Dental Service (Folktandvården Stockholm, Sweden), after approval by the Swedish Ethical Review Authority (Dnr 2019-04736). All methods were performed in accordance with the relevant guidelines and regulations. Informed consent was waived by the Swedish Ethical Review Authority.

### 2.1. Data Collection

**Figure 1:**
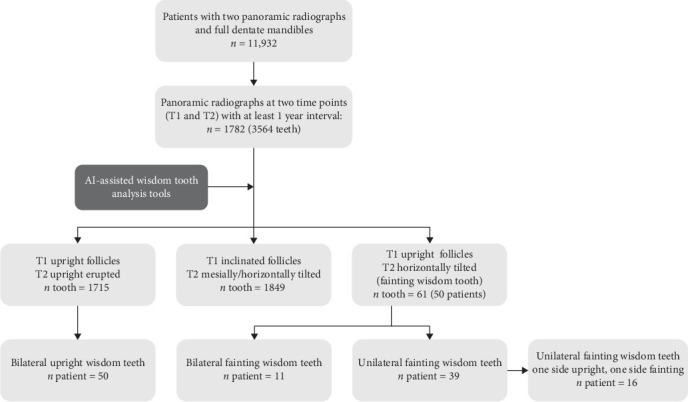
Flow diagram of the patient selection process.

From an original pooled database of 11,932 patients [6, 11], strict inclusion and exclusion criteria were applied to identify the panoramic radiographs of patients developing the rarely described fainting wisdom tooth phenomenon in fully dentate mandibles, as detailed in Table 1. This process resulted in the identification of 50 cases with fainting wisdom teeth, and additional 50 cases with initially upright follicles and vertically erupted wisdom teeth were selected to serve as controls, with the selection process illustrated in Figure 1. All cases identified derived from the electronic dental record system (T4 Practice Management Software; Carestream Dental; Atlanta; GA; USA) at Folktandvården Stockholm (Sweden). The patients included were referred for panoramic radiography for orthodontic treatment. Panoramic radiographs were acquired using the same X-ray machine (Planmeca ProMax 2D, Helsinki, Finland) by trained technicians following a standard protocol. The images, along with patients' age and gender information, were extracted anonymously using Romexis software (version 3.2.0; Planmeca, Helsinki, Finland).

Each patient had two panoramic radiographs, with the first taken between the ages of 8 and 15 (T1) and the second acquisition between the ages of 16 and 23 (T2). According to a study by Jung and Cho [12], the initial mineralisation of third molars occurs around 8 years of age, with crown completion typically around 15 years of age and apex closure occurring by approximately the age of 22. Selecting patients aged 8–15 years at T1 allows the observation of early stages of wisdom tooth development, while the age range of 16–23 years at T2 captures the critical period of eruption and any associated angular changes.

In the present study, an AI-assisted panoramic analysis using a DL model was used to identify the included cases. It facilitated the automated analysis and selection of the fainting wisdom tooth cases by measuring the angulation of the wisdom tooth relative to the second molar across two time points (T1 and T2), thus offering enhanced objectivity, reproducibility and efficiency over manual methods. The DL-based tool was developed by Vranckx et al. [7] by integrating functionalities into the open-source software LabelMe (https://github.com/wkentaro/labelme), and has been technically and clinically validated [6, 7]. This study involved a dataset of 838 patients, comprising 1676 wisdom teeth at various stages of development within the same age range as the samples used in the present study. The dataset covered all eruption positions, from upright to horizontal. The model was a fully CNN (ResNet-101 backbone), and demonstrated accuracy of 98.1% of angulations within ±5° error and an IoU of 90%.

A subsequent study by Chopra et al. [6] successfully employed the AI tool on a dataset of 771 patients (1542 wisdom teeth), further demonstrating the AI's generalisability across different populations. Wisdom tooth eruption prediction is optimised through automation of the angulation measurement procedure, as the AI tool can outperform the manual method by 2–4 times. The automated network achieved an accuracy of 79.7% (with an error threshold of 2.50°) and 98.1% (with an error threshold of 5.0°). Importantly, the tool functioned as previously validated, with no adjustments made for the present study. The present AI tool was considered as a clinical decision-support tool, meanwhile facilitating the measurements and decreasing the human measurement variability [6, 7]. Its use aligns with current recommendations for AI transparency and reporting in medical imaging, as outlined in the checklist for AI in medical imaging (CLAIM) [13].

**Figure 2:**
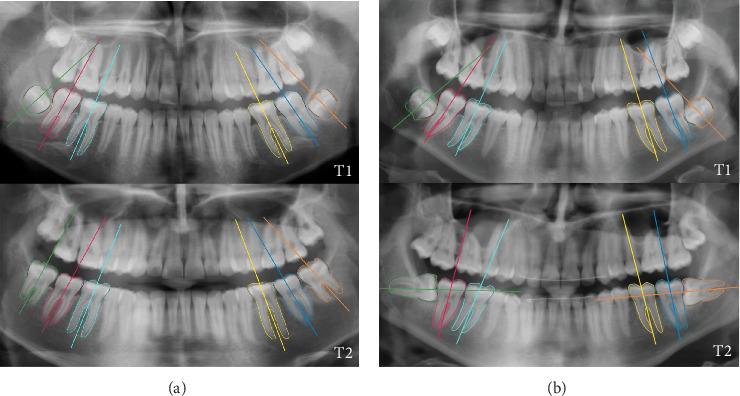
Consecutive panoramic radiographs of two subjects at two different exposure times (T1 and T2). (a) Vertically erupted mandibular wisdom teeth at T2 as predicted based on the angulation at T1. (b) Horizontal angular change of mandibular wisdom teeth at T2 illustrating the 'fainting wisdom tooth'.

The subset of included cases showed an upright wisdom tooth follicle at T1, with a mesial to horizontal tilting at T2. This mesial to horizontal tilting was identified when the wisdom tooth angulation was at least 75° at T2 (Figure 2). Prior to inclusion in the current dataset, all panoramic radiographs fitting the AI-assisted selection were further carefully inspected by an oral and maxillofacial radiologist. In case of doubt, a consensus was sought with a senior oral and maxillofacial radiologist. A simplified version of Demirjian's classification was used to describe the development stage of wisdom teeth: follicles without root formation (stages A–D), wisdom teeth starting root or bifurcation formation (stage E) and wisdom teeth with root length equal to or greater than the crown height (stages F–H) [14].

### 2.2. Study Variables

**Figure 3:**
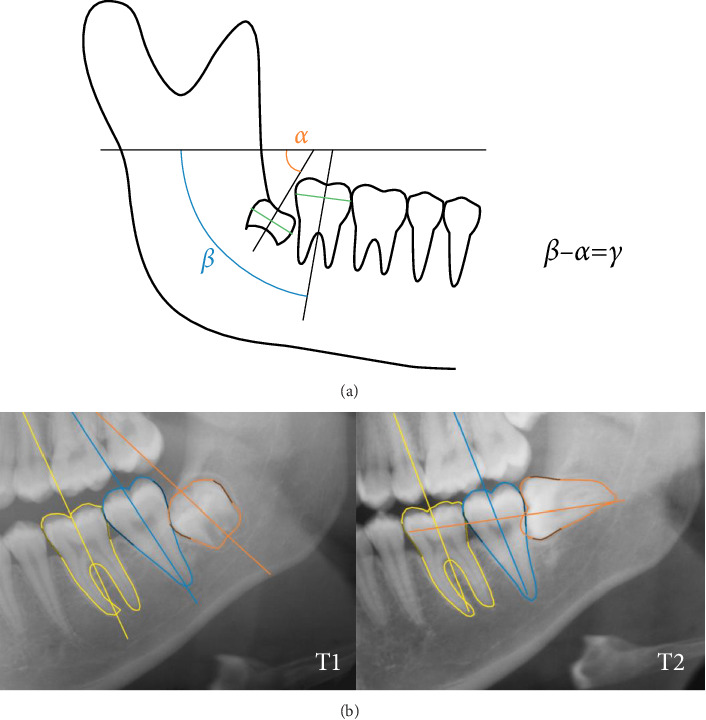
Wisdom tooth angulation measurement with AI-assisted mandibular wisdom tooth prediction tool [7]. (a) M2 and wisdom tooth angulation were assessed in relation to horizontal reference line. Wisdom tooth angulation (*γ*) defined by calculating the difference between M2 angle (*β*) and wisdom tooth angle (*α*). (b) A case of fainting wisdom tooth with molar segmentation maps and orientation lines by AI tool showing angulation of at least 75° at T2.

**Figure 4:**
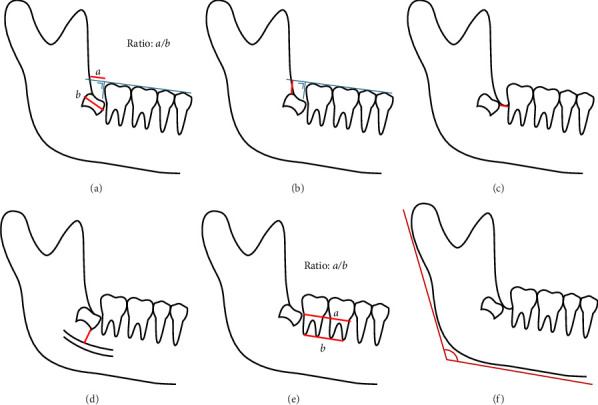
Study variables: (a) space/wisdom tooth crown width ratio, (b) wisdom tooth depth, (c) wisdom tooth proximity to second molar, (d) wisdom tooth proximity to the mandibular canal, (e) CEJ/apical width ratio of first and second molar and (f) gonial angle.

A thorough quantitative analysis was conducted to explore potential risk factors associated with this phenomenon. The study variables were assessed as follows:

1. Wisdom tooth angulation was calculated using a DL-based automated mandibular molar angulation measurements. Initially, the tooth angulation line was defined by dividing the crown into two equal halves and drawing a line through the midpoint, representing the widest diameter of the crown. The AI tool then calculated the final angle of the wisdom tooth by assessing the difference between the angles of the second molar (M2) and wisdom teeth as described in Vranckx et al. [7] and Chopra et al. [6] (Figure 3).
2. The space/wisdom tooth crown width ratio was defined by measuring the space between the distal contact point of M2 and the anterior border of ramus and divided by the wisdom tooth crown width (Figure 4A).
3. The wisdom tooth depth was measured from the occlusal plane to the most superior part of the tooth (Figure 4B).
4. The wisdom tooth proximity to the second molar was measured between the most mesial part of wisdom tooth and the distal part of M2. A negative result indicates that wisdom tooth overlaps with M2. (Figure 4C).
5. The wisdom tooth proximity to the mandibular canal was measured between the most inferior part of wisdom tooth and the adjacent superior wall of mandibular canal. A negative result indicates that wisdom tooth overlaps with mandibular canal. (Figure 4D).
6. The cemento-enamel junction (CEJ)/apical width ratio of first molar (M1) and M2 was defined by measuring the distance from the CEJ on the mesial aspect of M1 to the CEJ on the distal aspect of M2, divided by the distance from the most apical mesial point of the M1 mesial root to the most apical distal point of the M2 distal root (Figure 4E).
7. The gonial angle was measured by evaluating the angle formed between the tangent line to the posterior border of condyle and ramus and tangent line to lower mandibular border (Figure 4F).

The linear measurements results were further normalised with the mesial-distal CEJ width of the first molar, as observed in each T1 and T2 panoramic radiograph.

### 2.3. Statistical Analysis

All statistical analyses were conducted using R (version 4.3.1, R Core Team, Vienna, Austria) in conjunction with RStudio (2023.06.1 + 524). The Shapiro–Wilk test was employed to assess the normality of data distribution for each variable. Baseline characteristics of patients and all observed parameters were compared using the independent *t*-test for normally distributed data and the Mann–Whitney *U*-test for data that were not normally distributed. For the categorical variable of gender, comparisons were performed using the chi-square test. These statistical tests facilitated comparisons between the fainting group and the control group. A *p*-value threshold of 0.05 was set for determining statistical significance.

**Figure 5:**
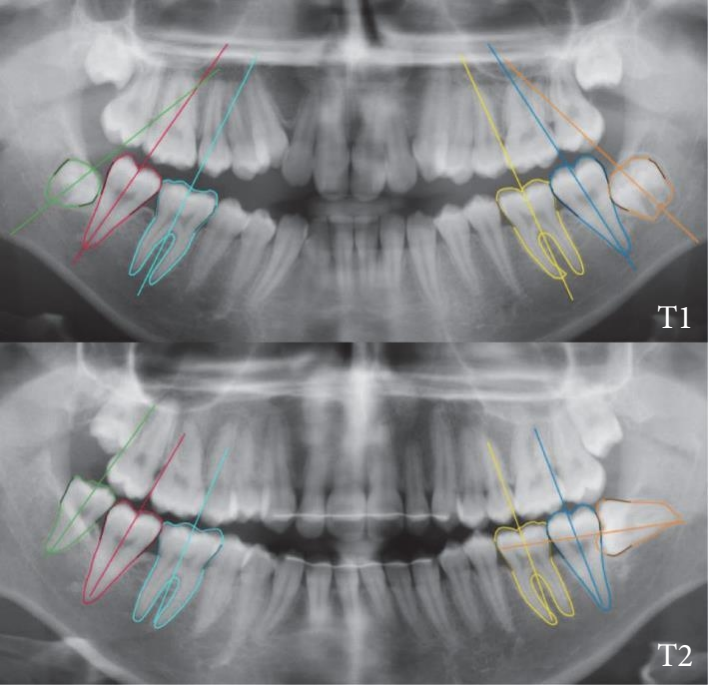
Consecutive panoramic radiographs of a patient with unilateral fainting wisdom tooth: (T1) upright wisdom tooth germs on both sides and (T2) vertically erupted wisdom tooth on right side and fainting wisdom tooth on left side.

Logistic regression analyses were conducted to identify significant factors associated with the occurrence of fainting wisdom tooth, with a significance value set at *p*  < 0.05. The first analysis evaluated how changes in variables between time points T2 and T1 influenced the likelihood of fainting wisdom tooth. The variables included age, space/wisdom tooth crown width ratio, wisdom tooth depth, proximity to the mandibular canal, proximity to the second molar, CEJ/apical width ratio of M1 and M2 and gonial angle. This analysis aimed to understand the impact of tooth eruption progression on the outcome. A second logistic regression analysis was performed to determine whether the initial position of the wisdom tooth at T1 influenced the risk of fainting wisdom tooth. Additionally, a within-subject comparison analysis was conducted for cases with unilateral fainting molars, as illustrated in Figure 5, which shows a patient with upright wisdom tooth germs on both sides at T1, later presenting with a vertically erupted wisdom tooth on one side and a fainting wisdom tooth on the other side at T2.

## 3. Results

A total of 50 cases exhibiting the fainting wisdom tooth pattern were identified through AI-assisted analysis on panoramic radiographs, corresponding to a prevalence of approximately 0.4% across the pooled data. As shown in Table 2, this group comprised 64% of males (*n* = 32) and 36% of females (*n* = 18). The median age at the time of the initial panoramic radiograph (T1) was 14 years. The median angulation of the fainting wisdom teeth at T1 was 29°. In comparison, the control group consisting of 50 cases with upright erupted wisdom teeth, had a median age of 14 years and a median wisdom teeth angulation of 18° at T1.

Table 3 summarises the comparative analyses between the 50 cases of fainting wisdom teeth and 50 control cases with upright-positioned wisdom teeth. The angulation of fainting wisdom tooth increased from T1 to T2, exhibiting a significantly different pattern compared to the control group. Significant differences (*p* < 0.05) were observed in the following variables: space/wisdom tooth crown width ratio, tooth depth in the jaw, proximity to second molar, CEJ/apical width ratio of first and second molar and gonial angle.

The fainting group exhibited a significantly greater increase in wisdom tooth angulation, with a median change from 29° at T1 to 83° at T2, compared to the control group's change from 18° at T1 to 20° at T2 (*p* < 0.001). At T1, the space-to-wisdom tooth crown width ratio was lower in the fainting group. However, the control group showed a significantly higher increase in this ratio from T1 to T2 (*p*=0.020), implying that increased space over time might facilitate an upright positioning, reducing the likelihood of fainting. Additionally, at T1, the fainting group had tooth follicles positioned closer to the occlusal plane compared to the control group. In contrast, the control group demonstrated significantly more tooth movement towards the occlusal plane during the eruption process from T1 to T2 (*p* < 0.001), suggesting that increased follicular depth within the jaw might favour an upright eruption pathway.

Proximity to the mandibular canal was similar between the groups. However, proximity to the M2 differed significantly, with the fainting group showing more contact with M2 compared to the control group (*p*=0.003). This closer contact with M2 suggests that spatial restrictions from the adjacent tooth might hinder wisdom tooth movement towards upright eruption. The M1-M2 CEJ/apical width ratio at T1 was higher in the fainting group and showed a greater increase from T1 to T2 (*p* < 0.001). This increased ratio suggests that the apical width is decreasing relative to the CEJ width, indicating that constrained space at the crown level might lead to reduced space for upright wisdom tooth eruption. Lastly, the gonial angle showed significantly different patterns between T1 and T2, with the fainting group exhibiting increased angulation while the control group showing a decrease (*p* < 0.001).

The logistic regression analysis of T2-T1 changes revealed several significant factors associated with the risk of fainting wisdom teeth, as detailed in Table 4. Wisdom tooth depth change had the most substantial impact, with an odds ratio (OR) of 0.1 (*p* < 0.001), indicating a strong inverse relationship, in which deeper wisdom teeth are less likely to cause fainting. Other significant factors that associated with the increased risk of fainting included a smaller increase in space/wisdom tooth crown width ratio (OR 1.6, *p*=0.02), a greater increase in proximity to the second molar (OR 1.8, *p*=0.013), a greater increase in the CEJ/apical width ratio of the first and second molar (OR 0.1, *p* < 0.001) and an increasing gonial angle (OR 0.5, *p* < 0.001).

In a separate analysis of variables measured at T1 (Table 5), logistic regression was used to determine any potential correlation between the initial state of wisdom teeth at T1 and the risk of fainting. Similar to the previous analysis, the wisdom tooth depth stood out as the most prominent factor, with an OR of 3.3 (*p* < 0.001), emphasising its crucial role in the likelihood of fainting wisdom tooth. Greater risk of fainting wisdom tooth was also expected in wisdom tooth with larger initial angulation (OR 0.9, *p* < 0.001), lower space/wisdom tooth crown width ratio (OR 2.4, *p* < 0.001), greater proximity to second molar (OR 2.4, *p* < 0.001) and higher CEJ/apical width ratio of first and second molar (OR 0.5, *p*=0.001).

Among the 50 fainting cases, 16 patients exhibited fainting wisdom teeth unilaterally, with an upright positioned wisdom tooth on the contralateral side. Eleven cases demonstrated bilateral fainting molars within the mandible, while the remaining cases presented a fainting molar on one side and an impacted wisdom tooth on the other side. Subsequently, split-mouth analysis was performed among the 16 cases with unilateral fainting molar occurrences and an upright-positioned wisdom tooth on the contralateral side (Table 6). Significant differences (*p* < 0.05) between the left and right sides were observed only in the proximity of the wisdom tooth to the occlusal plane, indicating that variations in wisdom tooth depth within the jawbone may have the greatest influence on the occurrence of fainting wisdom tooth.

## 4. Discussion

Several studies have been conducted to predict the eruption pattern of wisdom teeth; however, there remains a lack of longitudinal data recording the temporal changes in wisdom tooth angulation [6, 11]. This present study is unique as it provides longitudinal follow-up data, tracking the entire dataset over time, which allows for a detailed view of the changes in wisdom tooth eruption progress. Unlike many longitudinal studies that struggle with incomplete data due to the removal of third molars during the follow-up period, this study managed to include patients with all 16 mandibular teeth present at both time points.

Out of the initial pooled database of 11,932 patients, only 50 cases of fainting wisdom teeth were identified, indicating an estimated prevalence of 0.4%. This extremely low prevalence highlights the rarity of the condition and reflects the challenges encountered in rare condition research, where small sample sizes are common due to limited case availability. Hee et al. [15] and Marçal et al. [16], for example, conducted studies on rare conditions with samples of fewer than 50 cases. Despite this limitation, the present study provided reliable estimates with adequately narrow confidence intervals, demonstrating the feasibility of investigating rare conditions when analyses focus on effect sizes and precision.

The result reveals that wisdom tooth depth was identified as a key factor, showing a significant inverse relationship to the risk of fainting. These findings suggest that wisdom teeth with greater depth, which tend to exhibit more movement, are less likely to cause fainting. In accordance with this finding, Baik et al. [17] suggested that the initial depth of wisdom tooth can affect the possibility of vertical eruption, where the impacted group had a shorter distance from the occlusal plane. Furthermore, the failure rate of tooth eruption was found to be greater in a tooth that had a less shallow position [18].

The fainting group showed an initial upright position at T1, with mean angulation of 29°, which according to Nance et al. [8], considered favourable for upright eruptions. However, this group later deviated from the predicted pattern, tilting fully mesially and horizontally. Compared to control group, the fainting group showed significantly greater initial angulation. This finding aligns with previous studies indicating that a lower initial angulation is important for facilitating a proper eruption and alignment of the wisdom tooth [11, 19]. Nance et al. [8] reported that a wisdom tooth with angulation greater than 35° had only 3% chance of erupting to the occlusal plane after 2 years of follow-up. While Vranckx et al. [11] identified a lower initial threshold angle of 27°.

Insufficient retromolar space also emerged as a crucial predictor of fainting. The upright group showed a significantly higher space/wisdom tooth crown width ratio compared to the fainting group (*p*  < 0.05), consistent with Jakovljevic et al. [20]. Previous studies demonstrated that each millimetre increase in eruption space reduces impaction risk by 29%, while each degree improvement in angulation reduces risk by 11% [21]. This aligns with our findings showing the fainting group had greater initial angulation and smaller space ratios, indicating restricted retromolar space [22]. Additionally, closer proximity to the second molar was associated with fainting risk, both initially and increasing over time. Richardson [23] observed that such proximity creates obstacles along the eruption path, hindering vertical movement. The higher CEJ/apical ratio of adjacent molars in the fainting group further suggests constrained crown-level space, potentially leading to less retromolar space, increasing the risk of fainting wisdom tooth [21].

This present study also revealed intriguing findings on the gonial angle changes. At T1, the fainting group showed a lower gonial angle compared to the control group. However, the group, which did not have impacted teeth, demonstrated a decrease in the gonial angle at T2, consistent with previous studies indicating that gonial angle tends to decrease with age progression [20, 24]. In contrast, the fainting group showed an unexpected increase. This atypical increase could be related to muscular imbalance or weakness, possibly due to oral habits such as mouth breathing or thumb sucking, which are associated with vertical craniofacial morphology and altered mandibular growth patterns [25–27]. However, no functional or habit-related data were collected in this study, and these explanations remain hypothetical. Future research should investigate these potential associations further.

Demirel and Akbulut [2] suggested that a high gonial angle is indicative of a dolichofacial pattern, characterised by vertical facial growth, which poses a higher risk of impacted teeth, as indicated by Breik and Grubor [28]. Notably, individuals with a dolichofacial pattern exhibit less growth potential, resulting in less remodelling resorption of the anterior border of the ramus, and thus providing insufficient space for upright eruption of the wisdom tooth [28, 29]. This finding aligns with the previously described lower space/wisdom tooth crown width ratio in the fainting group.

A split-mouth analysis comparing fainting and upright sides within the same patients was conducted, avoiding bias regarding inter-patient variability such as age, gender and genetic factors. Interestingly, initial wisdom tooth angulation showed no significant difference between sides, suggesting unilateral angular changes occur within patients. However, the fainting side demonstrated significantly closer proximity to the occlusal plane. While space/wisdom tooth width ratio and CEJ/apical ratios for first and second molars showed no significant differences, the fainting side appeared more anatomically constrained. These findings suggest that proximity to the occlusal plane and limited eruption space may prevent optimal upright positioning and contribute to fainting episodes rather than initial angulation [23].

Genetic factors are believed to play a significant role in wisdom tooth positioning. A previous study focusing on monozygotic (MZ) twins described a significant genetic effect on the normal course of wisdom tooth development and eruption with relatively minor environmental contribution [30]. In line with this finding, Isomura et al. [31] stated that the degree of eruption was more similar among MZ twins than dizygotic (DZ) twins. However, it is noteworthy that the degree of third molar eruption was not always identical, even in MZ twins, implying that environmental factors also contribute to the tooth eruption. Nonetheless, the evidence suggests that genetic factors are likely to have a more significant influence than environmental factors [30, 31].

Observing the positional traits of wisdom teeth is essential in clinical practice, as it can guide preventive measures to avoid the fainting phenomenon. Factors such as wisdom tooth initial position in the jaw and available eruption space suggest that monitoring these aspects could facilitate timely intervention. Early identification of patients at-risk through periodic radiographic assessments could inform preventive strategies. These strategies include prophylactic extraction before complete root formation when fainting patterns are predicted, orthodontic space management to increase retromolar space, more frequent radiographic monitoring for high-risk cases and early intervention when initial mesial tilting is observed. These measures could minimise the complications associated with fainting molars, such as impaction, crowding or damage to adjacent teeth [32].

This study demonstrates how AI can enable research on rare clinical phenomena that would otherwise be logistically challenging. By automating measurements across 11,932 panoramic radiographs, we could identify and analyse 50 cases of this rare eruption pattern, a task that would have been impractical using manual methods alone. This approach exemplifies how AI can support, rather than replace, clinical research by handling large-scale data processing while maintaining clinical decision-making and interpretation in human hands. The combination of AI-assisted measurement and clinical expertise allowed for comprehensive analysis of multiple anatomical parameters over time.

The strength of this study lies in analysing a large cohort with longitudinal panoramic radiographs, ideal for identifying rare eruption patterns. However, the retrospective design presents limitations including selection bias from all subjects undergoing orthodontic treatment, inability to assess clinical factors like oral habits and myofunctional aspects, and lack of documentation of eruptive complications (such as soft tissue inflammation, mechanical obstruction or pericoronitis) that might influence fainting episodes. Additionally, panoramic radiographs have inherent limitations of two-dimensional superimposition, enlargement and deformation. Future prospective studies should include non-orthodontic controls, monitor eruptive complications to understand mechanisms driving directional changes and utilise three-dimensional imaging for more detailed analysis of wisdom tooth development over time.

## 5. Conclusion

The present study is the first to describe the rare fainting wisdom tooth phenomenon, observed in 0.4% of fully dentate mandibles. This condition is characterised by a transition of the mandibular wisdom tooth from an initial upright position to a fully mesial and horizontal inclination upon eruption. Using AI-assisted longitudinal analysis of panoramic radiographs, several contributing factors were identified, including the initial position of the wisdom tooth and further changes in the eruption trajectory. In addition, anatomical constraints surrounding the developing wisdom tooth may hinder vertical eruption, increasing the likelihood of fainting.

## Figures and Tables

**Table 1:** Inclusion and exclusion criteria for included panoramic radiographs.

Criteria	Fainting	Control
Inclusion		
1. Minimum 1 year interval between T1 and T2 radiograph	✓	✓
2. Fully dentate mandible	✓	✓
3. Upright unerupted wisdom tooth follicle at T1	✓	✓
4. Mesioanguler to horizontally erupted wisdom tooth at T2	✓	✗
5. Vertically erupted wisdom tooth at T2	✗	✓
6. Second molar eruption stage reaching the occlusal plane and at least last stage of apical root formation at T1 radiograph	✓	✓
7. Panoramic radiographs with good contrast, high image definition and no distortion, artefacts or positioning errors	✓	✓
Exclusion		
1. Mandibles with missing or supernumerary teeth, pathologies, maxillofacial trauma or surgery and craniofacial anomalies	✓	✓

**Table 2:** Descriptive data from the patients with fainting wisdom teeth as opposed to the control patients (median value).

Variables		Fainting (*n* = 50)		Control (*n* = 50)	
		T1	T2	T1	T2
Sex, *n* (%)	Male	32 (64)		27 (54)	
	Female	18 (36)		23 (46)	
Orthodontics referral, *n*		50		50	
Age (years)		13.6	19.5	13.7	19.1
Wisdom tooth development stage, *n*	No root formation	27	0	25	0
	Starting root formation	23	0	25	0
	2/3 Or fully developed root	0	50	0	50
Wisdom tooth angle (°)		28.6	82.9	17.6	1.9
Space/wisdom tooth crown width ratio		0.5	0.4	0.7	0.8
Wisdom tooth depth		0.2	0.1	0.4	0.0
Proximity to mandibular canal		−0.1	−0.1	0.0	0.0
Proximity to M2		−0.1	−0.1	0.02	0.0
M1-M2 CEJ/apical ratio		1.1	1.3	1.0	1.1
Gonial angle (°)		122.9	123.5	124.5	121.5

**Table 3:** Comparison of T2-T1 measurement changes in panoramic radiographs of mandibles with fainting wisdom teeth and those with upright erupting wisdom teeth.

Variables	Fainting	Control	*p*-Value
	*∆* (Range)	*∆* (Range)	
Sex, *n* (%)			
Male	32 (64)	27 (54)	0.312^a^
Female	18 (36)	23 (46)	
Age (years)	5.5 (2.2–9.9)	5.2 (2.2–9.9)	0.117^b^
Wisdom tooth angle (°)	34.8 (−51.4–95.68)	−17.4 (−51.4–56.2)	<0.001*⁣*^*∗*^^b^
Space/wisdom tooth crown width ratio	0.04 (−0.5–0.6)	0.1 (−0.2–0.6)	0.020*⁣*^*∗*^^c^
Wisdom tooth depth	−0.2 (−0.4–0.3)	−0.4 (−0.8–0)	<0.001*⁣*^*∗*^^b^
Proximity to mandibular canal	0.0 (−0.3–0.2)	0.0 (−0.3–0.4)	0.725^c^
Proximity to M2	−0.1 (−0.3–0.4)	−0.02 (−0.3–0.2)	0.003*⁣*^*∗*^^b^
M1-M2 CEJ/apical ratio	0.1 (−0.1–0.5)	0.02 (−0.2–0.2)	<0.001*⁣*^*∗*^^b^
Gonial angle (°)	0.9 (0.2–1.8)	−2.7 (−10.5–1.86)	<0.001*⁣*^*∗*^^b^

^a^Chi-square test.

^b^Independent *T*-test.

^c^Mann–Whitney *U*-test.

*⁣* ^*∗*^ *p* < 0.05.

**Table 4:** Logistic regression analysis to assess the association between changes in variables (*Δ*T2-T1) and the risk of fainting wisdom teeth at T2.

Variables	Univariate analysis		
	*p*-Value	OR	95% CI
Age	0.147	0.9	0.7–1.1
Space/wisdom tooth crown width ratio	0.02*⁣*^*∗*^	1.6	1.1–2.5
Wisdom tooth depth	<0.001*⁣*^*∗*^	0.1	0.05–0.3
Proximity to mandibular canal	0.722	1.1	0.7–1.6
Proximity to M2	0.013*⁣*^*∗*^	1.8	1.1–2.8
M1-M2 CEJ/apical ratio	<0.001*⁣*^*∗*^	0.1	0.04–0.3
Gonial angle	<0.001*⁣*^*∗*^	0.5	0.4–0.7

Abbreviations: CI, confidence interval; OR, odds ratio.

*⁣* ^*∗*^ *p* < 0.05.

**Table 5:** Logistic regression analysis to assess association between variables at T1 and the risk of fainting wisdom teeth at T2.

Variables	*p*-Value	OR	95% CI
Age	0.713	1.1	0.8–1.5
Sex			
Male	Reference	—	—
Female	0.310	1.5	0.7–3.4
Wisdom tooth angulation	<0.001*⁣*^*∗*^	0.9	0.9–1.0
Space/wisdom tooth crown width ratio	<0.001*⁣*^*∗*^	2.4	1.3–4.4
Wisdom tooth depth	<0.001*⁣*^*∗*^	3.3	1.9–5.8
Proximity to mandibular canal	0.060	1.5	1.0–2.3
Proximity to M2	<0.001*⁣*^*∗*^	2.4	1.4–4.0
M1-M2 CEJ/apical ratio	0.001*⁣*^*∗*^	0.5	0.3–0.7
Gonial angle	0.053	1.1	0.9–1.2

Abbreviations: CI, confidence interval; OR, odds ratio.

*⁣* ^*∗*^ *p* < 0.05.

**Table 6:** Non-parametric paired analysis of fainting and uprighting wisdom teeth based on T1 panoramic radiographic characteristics (*n* = 16).

Variables		A within-patient comparison of fainting versus erupting wisdom tooth		
		Fainting	Upright	*p*-Value
Side, *n* (%)	Left	7 (43.7)	9 (56.3)	—
	Right	9 (56.3)	7 (43.7)	
Wisdom tooth angulation, median (range)	—	30.0 (10.2–50.2)	25.1 (10.8–45.6)	0.255^a^
Space/wisdom tooth crown width ratio, median (range)	—	0.5 (0.3–0.9)	0.6 (0.4–0.9)	0.070^a^
M1-M2 CEJ/apical ratio	—	1.0 (0.9–1.4)	1.0 (0.9–1.3)	0.195^a^
Proximity to occlusal plane, *n*	Yes	12	4	0.008*⁣*^*∗*^^b^
	No	4	12	
Contact with mandibular canal, *n*	Yes	13	10	0.453^b^
	No	3	6	
Contact with M2, *n*	Yes	13	8	0.125^b^
	No	3	8	

^a^Wilcoxon Signed-rank test.

^b^McNemar test.

*⁣* ^*∗*^ *p* < 0.05.

## Data Availability

The data that support the findings of this study are available from the corresponding author upon reasonable request.
